# Synthesis and Preclinical Characterization of the PSMA-Targeted Hybrid Tracer PSMA-I&F for Nuclear and Fluorescence Imaging of Prostate Cancer

**DOI:** 10.2967/jnumed.118.212720

**Published:** 2019-01

**Authors:** Margret Schottelius, Alexander Wurzer, Katharina Wissmiller, Roswitha Beck, Maximilian Koch, Dimitrios Gorpas, Johannes Notni, Tessa Buckle, Matthias N. van Oosterom, Katja Steiger, Vasilis Ntziachristos, Markus Schwaiger, Fijs W.B. van Leeuwen, Hans-Jürgen Wester

**Affiliations:** 1 Chair for Pharmaceutical Radiochemistry, Technische Universität München, Garching, Germany; 2 Chair for Biological Imaging (CBI), Technische Universität München, Munich, Germany, and Institute for Biological and Medical Imaging (IBMI), Helmholtz Centre Munich, Oberschleißheim, Germany; 3 Interventional Molecular Imaging Laboratory, Department of Radiology, Leiden University Medical Center, Leiden, The Netherlands; 4 Institute for Pathology, Klinikum Rechts der Isar, Technische Universität München, Munich, Germany; and; 5 Department of Nuclear Medicine, Klinikum Rechts der Isar, Technische Universität München, Munich, Germany

**Keywords:** PSMA, prostate cancer, fluorescence, hybrid tracer, intraoperative guidance

## Abstract

The prostate-specific membrane antigen (PSMA)–targeted radiotracers ^68^Ga/^177^Lu-PSMA-I&T and ^99m^Tc-PSMA-I&S (for **i**maging and **s**urgery) are currently successfully used for clinical PET imaging, radionuclide therapy, and radioguided surgery of metastatic prostate cancer. To additionally exploit the high sensitivity and spatial resolution of fluorescence imaging for improved surgical guidance, a PSMA-I&T–based hybrid tracer, PSMA-I&F (DOTAGA-k(Sulfo-Cy5)-y-nal-k-Sub-KuE), has been developed and evaluated. **Methods:** The in vitro PSMA-targeting efficiency of PSMA-I&F, the reference PSMA-I&T, and their corresponding ^nat^Ga-/^68^Ga- and ^nat^Lu/^177^Lu counterparts was determined in LNCaP cells via competitive binding assays (IC_50_) and dual-tracer radioligand and fluorescence internalization studies. Biodistribution and small-animal PET imaging studies were performed in CB17 SCID and LNCaP xenograft–bearing SHO mice, respectively, and complemented by intraoperative far-red fluorescence imaging using a clinical laparoscope. Additionally, fully automated serial cryosectioning and fluorescence imaging of 1 tumor-bearing animal as well as PSMA immunohistochemistry and fluorescence microscopy of organ cryosections (tumor, kidney, spleen) were also performed. **Results:** Compared with the parent PSMA-I&T analogs, the PSMA affinities of PSMA-I&F and its ^nat^Ga-/^nat^Lu-complexes remained high and unaffected by dye conjugation (7.9 < IC_50_ < 10.5 nM for all ligands). The same was observed for the internalization of ^68^Ga- and ^177^Lu-PSMA-I&F. In vivo, blood clearance of ^68^Ga- and ^177^Lu-PSMA-I&F was only slightly delayed by high plasma protein binding (94%–95%), and very low accumulation in nontarget organs was observed already at 1 h after injection. Dynamic PET imaging confirmed PSMA-specific (as demonstrated by coinjection of 2-PMPA) uptake into the LNCaP xenograft (4.5% ± 1.8 percentage injected dose per gram) and the kidneys (106% ± 23 percentage injected dose per gram). Tumor-to-background ratios of 2.1, 5.2, 9.6, and 9.6 for blood, liver, intestines, and muscle, respectively, at 1 h after injection led to excellent imaging contrast in ^68^Ga-PSMA-I&F PET and in intraoperative fluorescence imaging. Furthermore, fluorescence imaging of tissue cryosections allowed high-resolution visualization of intraorgan PSMA-I&F distribution in vivo and its correlation with PSMA expression as determined by immunohistochemistry. **Conclusion:** Thus, with its high PSMA-targeting efficiency and favorable pharmacokinetic profile, ^68^Ga/^177^Lu-PSMA-I&F serves as an excellent proof-of-concept compound for the general feasibility of PSMA-I&T–based hybrid imaging. The PSMA-I&T scaffold represents a versatile PSMA-targeted lead structure, allowing relatively straightforward adaptation to the different structural requirements of dedicated nuclear or hybrid imaging agents.

Triggered by the introduction of ^68^Ga-PSMA-11 PSMA PET in 2012 (1) for the diagnosis and staging of prostate cancer (2,3), PSMA-targeted diagnostic imaging and subsequently developed therapeutic approaches (4–6) have become valuable new tools in the clinical management of prostate cancer (7).

The rapid success of this translational effort is based on the availability of powerful PSMA-targeted tracers for all relevant clinical applications (SPECT/PET/targeted radionuclide therapy) (7–9). Tracer development, in turn, has been facilitated by the relative tolerance of the most commonly used central PSMA binding motif, EuX (glutamate-urea-X, with X = lysine, glutamate, or cysteine), toward diverse and even bulky chemical modifications (1,4,10). This allows relatively straightforward adjustment of the tracer structure to the requirements for the respective labeling strategy (^99m^Tc, ^18^F, diagnostic and therapeutic M^3+^ radiometals) without compromising PSMA-targeting efficiency and has thus also promoted the rapid expansion of PSMA-targeted theranostics toward α-therapy using ^213^Bi (11,12) or ^225^Ac (13). Furthermore, the flexibility of EuX-based tracers toward modification also supports the implementation of chemical strategies for the fine-tuning of their pharmacokinetic profile (5,14,15) and the generation of suitable fluorescent probes (16–18).

Recently, the existing classic theranostic concept comprising diagnostic imaging and targeted radionuclide therapy has been expanded by the use of ^111^In-PSMA-I&T (19) and ^99m^Tc-PSMA-I&S (for **i**maging and **s**urgery) (20) for PSMA-targeted radioguided surgery of soft-tissue metastases in oligometastatic recurrent prostate cancer (21–23). A practical disadvantage of relying on intraoperative radioguidance alone is the acoustic and numeric surgical guidance provided by γ-probes, which, although highly sensitive, have a limited spatial resolution. As an alternative, the use of fluorescent tracers has been proposed (24). Although fluorescence imaging supports real-time visual image guidance, it suffers from severe attenuation in tissue and thus is not suitable to detect metastases in deeper-lying lymph nodes (25).

In this context, the use of multimodal, or rather, hybrid tracers that combine γ-emission and fluorescence in one (targeted) molecule merges the best of both modalities. The unquestionable utility of such a hybrid approach has initially been established by the introduction of the hybrid sentinel lymph node tracer ICG-^99m^Tc-nanocolloid (ICG = indocyanine green) (26) in different cancer types. The various prostate cancer–related studies performed with this tracer have demonstrated how a hybrid tracer design can help improve radioguidance procedures (27), thereby triggering the recent development of various dedicated hybrid nuclear/fluorescence probes (28–33).

Given the potential demonstrated by PSMA-targeted radioguided surgery, it is a logical next step to study whether this procedure, similar to sentinel lymph node biopsy procedures, can benefit from a hybrid fluorescent/nuclear guidance concept. First examples of PSMA-targeted hybrid tracers, either small-molecule inhibitors (31,32) or antibodies (33), have recently been evaluated preclinically and demonstrate the general feasibility of such an approach. Although most tracers for fluorescence-guided surgery include the commercially available near-infrared dye IRDye800CW (λ_ex_ [excitation wavelength] = 773 nm, λ_em_ [emission wavelength] = 792 nm), the far-red cyanine dye Cy5 (λ_ex_ = 640 nm, λ_em_ = 656 nm) has also been found to be of utility in surgical guidance (34,35). Furthermore, the superiority of Cy5 over the near-infrared dyes Cy7 (λ_ex_ = 760 nm, λ_em_ = 780 nm) and ICG (λ_ex_ = 800 nm, λ_em_ = 820 nm) with respect to detection sensitivity (0.05 vs. 3.15 μM for ICG), tissue penetration (9 vs. 6 mm for ICG), and brightness (quantum yield, 28% vs 0.3% for ICG) has recently been reported (36). Additionally, it was shown that the disulfonated analog of Cy5, Sulfo-Cy5 (λ_ex_ = 646 nm, λ_em_ = 662 nm), in addition to being substantially more hydrophilic, displayed even more favorable characteristics with respect to optical stability and brightness (51 × 10^3^ vs. 23 × 10^3^⋅M^−1^⋅cm^−1^ for Cy5) (37).

Sulfo-Cy5 was therefore selected as the fluorescent dye of choice for the implementation of a first-generation hybrid concept based on the PSMA-I&T scaffold (Fig. 1). Using this established backbone has the advantage of allowing an exact assessment of the influence of linker branching and dye conjugation on the pharmacokinetic properties of the novel tracer compared with the parent compounds ^68^Ga-PSMA-I&T and ^177^Lu-PSMA-I&T, based on already existing datasets (4). Here, we present the in-depth preclinical evaluation of the novel hybrid analog ^68^Ga/^177^Lu-PSMA-I&F (Fig. 1), with respect to PSMA-targeting efficiency and overall performance as a hybrid nuclear/fluorescent probe for the sensitive in vivo imaging of PSMA expression. Of course, the high-energy γ-emission of ^68^Ga-PSMA-I&F (and also of its ^177^Lu-labeled analog) severely challenges their clinical application in radio-/fluorescence-guided surgery, both with respect to spatial resolution during surgery and to patient dosimetry. It is important to note, however, that PSMA-I&F was specifically designed as a first proof-of-concept compound to demonstrate the general suitability of the underlying tracer design (PSMA-I&T–based) for the generation of tailored PSMA ligands for a broad spectrum of multimodal applications. Our previous experience with ^99m^Tc-PSMA-I&S has demonstrated the relative ease of adapting the labeling chemistry and pharmacokinetic profile of the PSMA-I&T scaffold to a dedicated application in radioguided surgery (20). Thus, a swift transfer of the insights gained in this proof-of-concept study to a next generation of corresponding ^99m^Tc-labeled Sulfo-Cy5-PSMA ligands with potential for a rapid transfer into the clinical/surgical setting may be anticipated.

**FIGURE 1.**
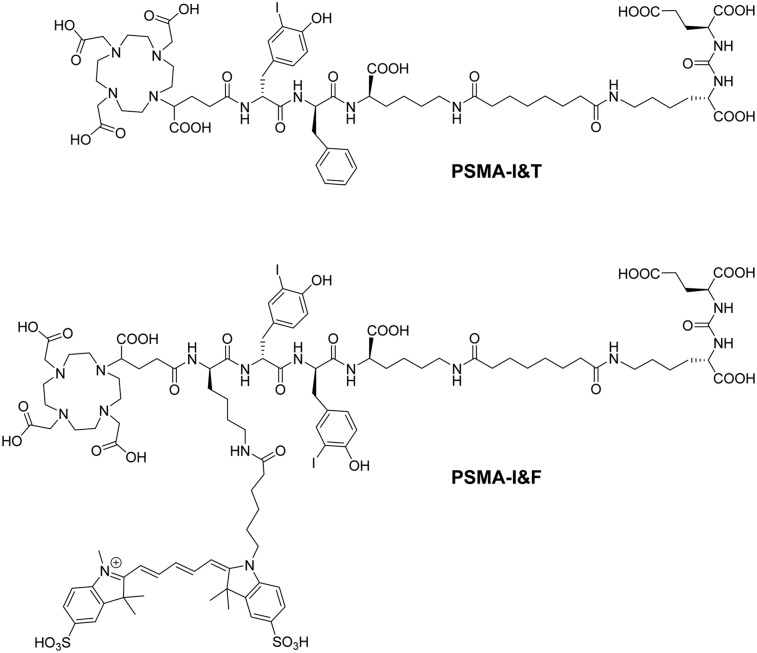
Structure of PSMA-I&T (4) and its Sulfo-Cy5–conjugated hybrid analog, PSMA-I&F.

## MATERIALS AND METHODS

### Precursor Synthesis and Radiolabeling

The synthesis of PSMA-I&F (DOTAGA-d-Lys(N_ε_-Sulfo-Cy5)-D-(3-iodo)Tyr-d-(3-iodo)Tyr-d-Lys(N_ε_-Sub-KuE), Fig. 1) was performed in analogy to a previously published protocol (38) (Supplemental Fig. 1; supplemental materials are available at http://jnm.snmjournals.org). The ^nat^Ga- and ^nat^Lu-complexes of PSMA-I&F were prepared by dissolving 200–500 μg of peptide either in 20 mM GaNO_3_ or LuCl_3_ in 0.01 M HCl to yield a final peptide concentration of 1 mM. Solutions were heated to 95°C for 30 min in a sealed tube, and the product solutions were used as such for the preparation of dilution series for binding or internalization studies. ^68^Ga labeling of PSMA-I&F (5 nmol, 800 μL 2.7 M HEPES) was performed as previously described (39) using an automated system (GallElut^+^; Scintomics), and labeling with ^177^LuCl_3_ (itm) was performed manually using a standard protocol (2 nmol precursor, 10% [v/v] 1 M NH_4_OAc) (40). The radiochemical purity of ^68^Ga-PSMA-I&F and ^177^Lu-PSMA-I&F was determined via radio–thin-layer chromatography using iTLC silica gel–impregnated chromatography paper (Agilent) and 2 different mobile phases (i.e., 0.1 M aqueous sodium citrate and a 1:1 [v/v] mixture of 1 M aqueous NH_4_OAc and MeOH). The radioiodinated reference ligand (^125^I-BA)KuE ((*S*)-1-carboxy-5-(4-(-^125^I-iodo-benzamido)pentyl)-carbamoyl)-l-glutamic acid) was prepared as previously described (38).

### Determination of Lipophilicity and Plasma Protein Binding

The lipophilicity of ^68^Ga- and ^177^Lu-PSMA-I&F was determined via a modified shake-flask method as previously described (38). Plasma protein binding of both tracers was determined using 2 alternative methods, that is, incubation in fresh human plasma and subsequent ultrafiltration (20) and a previously established gradient high-performance liquid chromatography method for the quantification of human serum albumin (HSA) binding (41,42).

### In Vitro Evaluation

Competitive binding experiments (IC_50_) were performed in analogy to a previously published protocol (38) using LNCaP cells and (^125^I-BA)KuE as a standard radioligand. Internalization kinetics of ^68^Ga-PSMA-I&F and ^177^Lu-PSMA-I&F (2 nM) were investigated in dual-tracer internalization assays using PSMA-expressing LNCaP cells and (^125^I-BA)KuE (0.1 nM) as an internal reference. To differentiate between membrane-bound and internalized activity, an acid wash (50 mM NaOAc in saline, pH 4.5) was performed prior to lysis of the cells (1N NaOH) and quantification of internalized activity (lysate) using a γ-counter. Data were corrected for nonspecific internalization in the presence of 10 μM 2-PMPA (2-(Phosphonomethyl)pentane-1,5-dioic acid; Tocris Bioscience) and normalized to the specific internalization observed for the radioiodinated reference compound in the same experiment. For interexeperimental normalization, ^68^Ga-PSMA-I&T and ^177^Lu-PSMA-I&T (2 nM) were reassayed under these conditions. For fluorescence microscopy, PSMA-positive LNCaP and PSMA-negative PC3 cells (seeded on glass cover slips) were incubated with the respective PSMA-I&F analog (100 or 25 nM) in the absence (total binding/internalization) or presence (nonspecific binding/internalization) of 10 μM 2-PMPA at 37 °C for 5 or 60 min. Hoechst 33342 (final concentration, 2 μg/ml) and LysoTracker Green (Life Technologies; final concentration, 100 nM) were added 2 min before the end of incubation. To end incubation, the cover slips were removed from the incubation medium, washed with phosphate-buffered saline, and fixed in 4% Histofix solution. Ligand internalization was visualized using a Keyence BZ-9000 fluorescence microscope equipped with Cy5 (620/60 nm excitation, 700/75 nm emission), DAPI-BP (370/50 nm excitation, 447/60 nm emission), and GFP-BP (472.5/30 nm excitation, 520/35 nm emission) filters.

### In Vivo Evaluation

All animal experiments were conducted in accordance with the German Animal Welfare Act (Deutsches Tierschutzgesetz, approval no. 55.2-1-54-2532-71-13).

#### Biodistribution Studies

The biodistribution of ^177^Lu-PSMA-I&F was investigated in non–tumor-bearing male CB17 SCID (severe combined immunodeficiency) mice. In the case of ^68^Ga-PSMA-I&F, biodistribution studies were performed using LNCaP xenograft–bearing SHO (SCID hairless outbred) mice. Mice were injected intravenously with ^177^Lu-PSMA-I&F (9.3 MBq) or ^68^Ga-PSMA-I&F (13.2 MBq; the amount of injected peptide was kept constant at 0.2 nmol/mouse in all experiments) under isoflurane anesthesia and were sacrificed at 1 h (^68^Ga-PSMA-I&F, ^177^Lu-PSMA-I&F) and 6 h (^177^Lu-PSMA-I&F) after injection (groups of *n* = 5, respectively). The organs of interest were dissected, and the activity concentration in weighed tissue samples was quantified using a 2480 Automatic γ-Counter (PerkinElmer).

#### PET Imaging

Small-animal PET imaging was performed on a Siemens Inveon small-animal PET system. Under isoflurane anesthesia, LNCaP xenograft–bearing SHO mice were injected intravenously with 0.19–0.25 nmol (2–7 MBq) of ^68^Ga-PSMA-I&F. For competition studies, 1 μmol of 2-PMPA (2-phosphonomethyl pentanedioic acid; 226 μg/mouse) was coadministered. Dynamic imaging was performed after on-bed injection for 90 min. Static images were acquired 1 h after tracer injection with an acquisition time of 15 min. Images were reconstructed as single frames using Siemens Inveon software, using a 3-dimensional ordered-subset expectation maximum algorithm without scatter and attenuation correction.

#### Ex Vivo Fluorescence Microscopy

LNCaP xenograft–bearing CB17 SCID mice were injected with 2 nmol of PSMA-I&F in 100 μL of phosphate-buffered saline via the tail vein. At 1 h after injection, mice were sacrificed and the tissues of interest were removed, embedded in Tissue-Tek (Sakura Finetek Europe B.V.), and frozen to −18°C. Cryosections (10 μm) were prepared using a CM1950 cryostat (Leica), and fluorescence microscopy was performed on a Keyence BZ-9000 fluorescence microscope equipped with a Cy5 filter. Images were processed using the BZ-9000 analyzer software.

#### Histopathology and PSMA Immunohistochemistry

Hematoxylin and eosin staining was performed on deparaffinized 2-μm sections of mouse tissues with Eosin and Mayer’s Haemalaun according to a standard protocol. Immunohistochemistry was performed using a BondMax RXm system (Leica; all reagents from Leica) with a primary antibody against PSMA (abcam; ab133579, diluted 1:100 in antibody diluent).

#### Whole-Body Cryosectioning and Fluorescence Imaging

An LNCaP xenograft–bearing SHO mouse (the same animal that had been used for the static PET scan 2 d previously) was injected with 2 nmol of ^nat^Ga-PSMA-I&F, sacrificed at 1 h after injection, embedded in a mixture of Tissue-Tek and black ink (7.41% v/v) and frozen. Whole-body cryosectioning and fluorescence imaging was performed using a Leica CM 3500 cryostat at 80-μm steps. Details on the fluorescence imaging protocol and the instrumentation are provided in the supplemental materials. The serial sectioning and imaging system was fully automated, using custom software implemented in LabView (National Instruments) (43). Postprocessing was performed using MATLAB (The MathWorks), and Amira (FEI Visualization Sciences Group) was used for cross-sectional and longitudinal visualization of the acquired data and for 3-dimensional representation of the fluorescence data.

#### Intraoperative Fluorescence Imaging

Fluorescence imaging (intraoperative/ex vivo) of LNCaP xenografts in CB17 SCID mice (1 h after injection) was performed using 2 different instrumental setups: a handheld digital microscope for Cy5 (Dino-Lite Edge AM4115T-DFRW; AnMo Electronics Corp.) and DinoCapture 2.0 software (AnMo Electronics Corp.); and a 0°Firefly laparoscope that is compatible with the surgical robotic da Vinci Si system (Intuitive Surgical Inc.), using a fluid light cable for target illumination (495 FR; KARL STORZ Endoskope GmbH&Co.KG) and a bandpass filter (Edmund Optics Inc.) to isolate the Cy5 emission. Images were captured from the unprocessed Firefly video feed. For instrumental details see supplemental data.

## RESULTS

### Synthesis and Radiolabeling

PSMA-I&F was synthesized via a mixed solid-phase/solution-phase synthetic strategy, with coupling of Sulfo-Cy5-COOH to the fully deprotected, DOTAGA-functionalized backbone as the last step. PSMA-I&F was obtained in greater than 96% purity (ultraviolet absorption at 214 nm) and 28% yield for dye conjugation.

Radiolabeling was performed using standard automated (^68^Ga) or manual (^177^Lu) procedures, yielding ^68^Ga-PSMA-I&F and ^177^Lu-PSMA-I&F in isolated radiochemical yields of 74% and 98%, respectively (decay-corrected). The specific activities of ^68^Ga-PSMA-I&F and ^177^Lu-PSMA-I&F were 61 and 55 GBq/μmol, respectively, and radiochemical purity as determined by radio–thin-layer chromatography was greater than 98% for both compounds.

### In Vitro Characterization

All in vitro data obtained for PSMA-I&F and its corresponding (radio)metal chelates are summarized in Table 1; data for the respective PSMA-I&T reference compounds (4) are included for comparison. Overall, linker extension and conjugation with Sulfo-Cy5 dye led to slightly increased lipophilicities of ^68^Ga- and ^177^Lu-PSMA-I&F compared with the PSMA-I&T parent compounds, resulting in stronger nonspecific binding to HSA. However, these structural modifications have no detectable influence on the PSMA affinity (IC_50_) of PSMA-I&F compared with PSMA-I&T (4,38). Furthermore, and as observed for PSMA-I&T, PSMA affinity of PSMA-I&F also remains entirely unaffected by (radio)metal chelation. The same applies to the internalization efficiency of ^68^Ga-PSMA-I&F and ^177^Lu-PSMA-I&F into LNCaP prostate carcinoma cells compared with ^177^Lu-PSMA-I&T, with all 3 compounds showing identical tracer internalization after a 1-h incubation time (37°C). Only ^68^Ga-PSMA-I&T displayed unexpectedly high internalization efficiency compared with the reference (^125^I-BA)KuE under these conditions.

**TABLE 1:** PSMA Affinities, Internalization, and Lipophilicity of Ga- and Lu-PSMA-I&T (4) and of Respective Unlabeled and Labeled PSMA-I&F Analogs

Ligand	IC_50_ (nM)	Corresponding radioligand	Specific internalization (% of reference)*	Lipophilicity (log P_OW_)	Plasma protein binding (%)^†^
PSMA-I&T	10.2 ± 3.5				
Ga-PSMA-I&T	9.3 ± 3.3	^68^Ga-PSMA-I&T	206 ± 16	−4.30	52.0 (84.2)
Lu-PSMA-I&T	7.9 ± 2.4	^177^Lu-PSMA-I&T	114 ± 8	−4.12	78.6 (82.1)
PSMA-I&F	10.3 ± 0.7				98.3
Ga-PSMA-I&F	10.5 ± 2.1	^68^Ga-PSMA-I&F	103 ± 9	−3.40	93.7 (94.0)
Lu-PSMA-I&F	9.6 ± 1.7	^177^Lu-PSMA-I&F	106 ± 2	−3.53	95.0 (98.9)

* Specific internalization (total internalization corrected by internalization in the presence of 10 μM 2-PMPA) of the reference compound (^125^I-IBA)KuE was determined in the same experiment (dual-tracer study) and used for data normalization. Data represent specific internalization at 1-h incubation time.

† Numbers represent HSA binding of the nonradioactive compounds determined via chromatography; numbers in parentheses represent plasma protein binding determined using fresh human plasma and the corresponding radiolabeled analogs.

### Fluorescence Microscopy of Ligand Internalization

**FIGURE 2.**
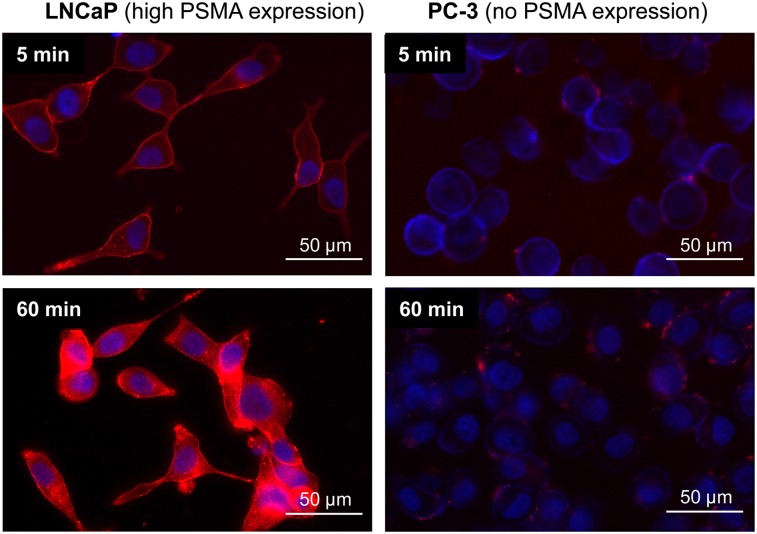
Fluorescence microscopy (overlay) of internalization of [^nat^Lu]PSMA-I&F (100 nM) into LNCaP prostate carcinoma cells after 5 (top left) and 60 (bottom left) min at 37°C. Nonspecific background internalization was determined using PSMA-negative PC-3 cells (right). Red fluorescence = Cy5 filter (PSMA-I&F); blue fluorescence = DAPI filter (Hoechst 33342).

The PSMA-mediated internalization of PSMA-I&F and its ^nat^Ga/^nat^Lu-analogs into LNCaP cells was also investigated using fluorescence microscopy (Fig. 2). As shown exemplarily for ^nat^Lu-PSMA-I&F in Figure 2, ligand binding and internalization are highly PSMA-specific, because virtually no background Cy5-fluorescence was detected for the PSMA-negative PC-3 cells under the same conditions. Although ^nat^Lu-PSMA-I&F fluorescence was primarily membrane-associated after 5 min, efficient ligand internalization into the cells was observed within 60 min at 37°C. At that time point, Cy5-fluorescence was found to almost entirely colocalize with LysoTracker Green, demonstrating rapid shuttling of ^nat^Lu-PSMA-I&F along the endosomal pathway into the lysosomes (Supplemental Fig. 3A). Furthermore, a comparative internalization assay demonstrated highly efficient and identical ligand internalization for PSMA-I&F, ^nat^Ga-PSMA-I&F, and ^nat^Lu-PSMA-I&F, even at lower ligand concentrations (25 nM) (Supplemental Fig. 3B), which is in accordance with the radioligand internalization data (Table 1).

### In Vivo Biodistribution Studies

Because the physicochemical and in vitro targeting characteristics of ^68^Ga- and ^177^Lu-PSMA-I&F were found to be nearly identical, both compounds were assumed to also display similar in vivo biodistribution profiles. Because of the longer half-life of ^177^Lu, which facilitates in vivo studies at later time points, ^177^Lu-PSMA-I&F was selected to determine the general biodistribution and clearance kinetics of PSMA-I&F–based ligands in non–tumor-bearing SCID mice at 1 and 6 h after injection (Table 2). Surprisingly, despite high plasma protein binding (Table 1), ^177^Lu-PSMA-I&F showed relatively fast background clearance within 6 h, with very low accumulation in the gastrointestinal tract and other nontarget organs. However, the PSMA-mediated tracer uptake in the kidneys was high and persistent.

**TABLE 2:** Biodistribution of ^177^Lu-PSMA-I&F and ^68^Ga-PSMA-I&F in CB17 SCID and LNCaP Xenograft–Bearing SHO Mice, Respectively (*n* = 4–5)

	^177^Lu-PSMA-I&F			
Organ	1 h after injection	6 h after injection	^68^Ga-PSMA-I&F, 1 h after injection	^68^Ga-PSMA-I&T, 1 h after injection
Blood	1.4 ± 0.2	0.29 ± 0.03	2.1 ± 0.4	0.5 ± 0.2
Heart	0.8 ± 0.1	0.24 ± 0.04	1.0 ± 0.1	0.3 ± 0.1
Lung	2.0 ± 0.3	0.81 ± 0.18	2.1 ± 0.5	1.5 ± 0.4
Liver	1.4 ± 0.5	0.58 ± 0.08	0.9 ± 0.1	1.0 ± 0.4
Spleen	14.6 ± 3.9	5.71 ± 0.60	12.8 ± 6.5	3.9 ± 1.5
Pancreas	0.8 ± 0.2	0.23 ± 0.04	0.7 ± 0.1	0.5 ± 0.2
Stomach	0.6 ± 0.1	0.28 ± 0.06	0.7 ± 0.1	0.4 ± 0.1
Intestines	0.4 ± 0.1	0.29 ± 0.10	0.5 ± 0.2	0.3 ± 0.1
Kidneys	76.9 ± 4.2	72.62 ± 5.88	105.8 ± 22.7	53.3 ± 9.0
Muscle	0.4 ± 0.1	0.11 ± 0.03	0.5 ± 0.1	0.4 ± 0.1
LNCaP tumor	—	—	4.5 ± 1.8	4.9 ± 1.6

Data are given in percentage injected dose per gram (%ID/g) and are mean ± SD. Data for ^68^Ga-PSMA-I&T from a previous publication (4) are included for comparison.

Although displaying a slightly higher blood concentration at 1 h after injection, ^68^Ga-PSMA-I&F showed an identical distribution pattern in almost all tissues, with the only exception of an increased renal uptake compared with ^177^Lu-PSMA-I&F. Uptake in the LNCaP xenograft was in the expected range (4,20), leading to reasonable tumor-to-background ratios of 2.1, 5.2, 9.6, and 9.6 for blood, liver, intestines, and muscle, respectively, at 1 h after injection.

### Small-Animal PET Imaging

**FIGURE 3.**
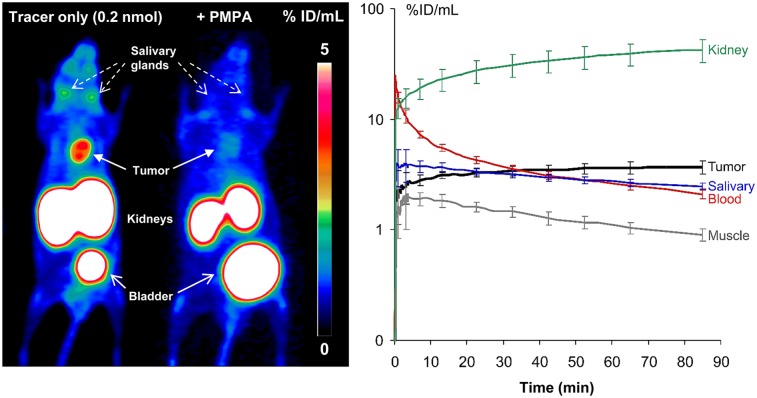
(Left) ^68^Ga-PSMA-I&F PET of LNCaP xenograft–bearing SHO mice (maximum-intensity projection, static scan, 1 h after injection) without (left mouse) or with (right mouse) coinjection of excess 2-PMPA. (Right) Time–activity curves for selected organs 0–90 min after injection of ^68^Ga-PSMA-I&F (0.2 nmol) in LNCaP xenograft–bearing SHO mouse.

That these target-to-nontarget ratios are sufficient for high-contrast imaging of PSMA-expressing tumors was confirmed by ^68^Ga-PSMA-I&F PET (Fig. 3). Furthermore, a competition study (coinjection of an excess of 2-PMPA) demonstrated the pronounced PSMA specificity of ^68^Ga-PSMA-I&F uptake in tumor and kidney and blockable tracer uptake in the salivary glands. A dynamic PET scan (0–90 min after injection) revealed fast background clearance kinetics (blood and muscle) with no indication of unwanted tracer retention as well as steadily increasing tracer accumulation in PSMA-expressing tissues (tumor, kidney) over time. Interestingly, ^68^Ga-PSMA-I&F showed no retention in the salivary glands but washout at a slightly slower rate than observed for muscle.

### Whole-Body Cryosectioning and Fluorescence Imaging

**FIGURE 4.**
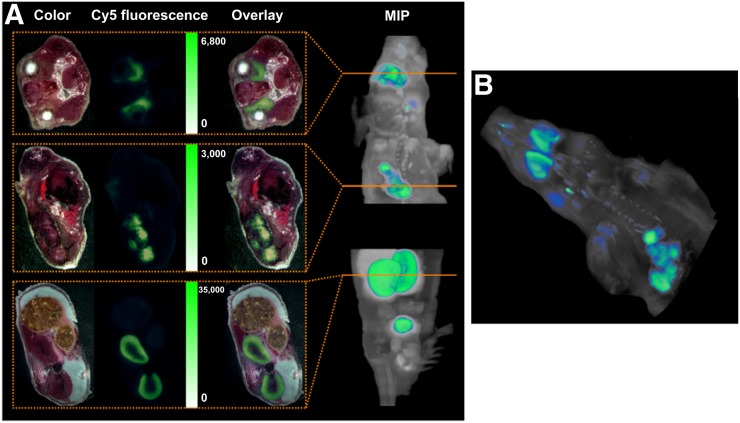
(A) Color and fluorescence images of whole-body cryosections of LNCaP xenograft–bearing SHO mouse 1 h after injection of 2 nmol ^nat^Ga-PSMA-I&F. (Top) Representative section of salivary glands. (Middle) Representative section of tumor region. (Bottom) Representative section of kidneys. Please note that images are scaled to same visual intensity. On right, the maximum-intensity projection (MIP) is at *xz* plane. Orange lines correspond to exact locations of representative sections shown to left. (B) Three-dimensional rendering of Sulfo-Cy5 fluorescence images obtained for consecutive 2-dimensional whole-body cryosections (as in B) of LNCaP xenograft–bearing SHO mouse 1 h after injection of 2 nmol ^nat^Ga-PSMA-I&F.

Results from fluorescence imaging (Cy5) of single whole-body cryosections 1 h after intravenous administration of ^nat^Ga-PSMA-I&F and subsequent 3-dimensional reconstruction of the 2-dimensional data are summarized in Figure 4. Fluorescence images show accumulation of ^nat^Ga-PSMA-I&F in the tumor, kidney, and salivary glands with relative fluorescence intensities that correlate closely with the respective tracer uptake observed in the PET imaging studies using ^68^Ga-PSMA-I&F (Fig. 3). Additionally, because of its superior spatial resolution and sensitivity, which surpasses that of nuclear imaging techniques by orders of magnitude, fluorescence imaging revealed considerable inhomogeneity of ^nat^Ga-PSMA-I&F uptake in the tumor xenograft as well as the restriction of renal ^nat^Ga-PSMA-I&F uptake to the kidney cortex. Furthermore, homogeneous accumulation in the salivary glands (Figs. 4A and 4B) and faint but discernible ligand uptake in PSMA-positive thoracic paravertebral ganglia, which has also been documented in humans (44–46), were observed (Fig. 4A).

### Immunohistochemistry and Fluorescence Microscopy

**FIGURE 5.**
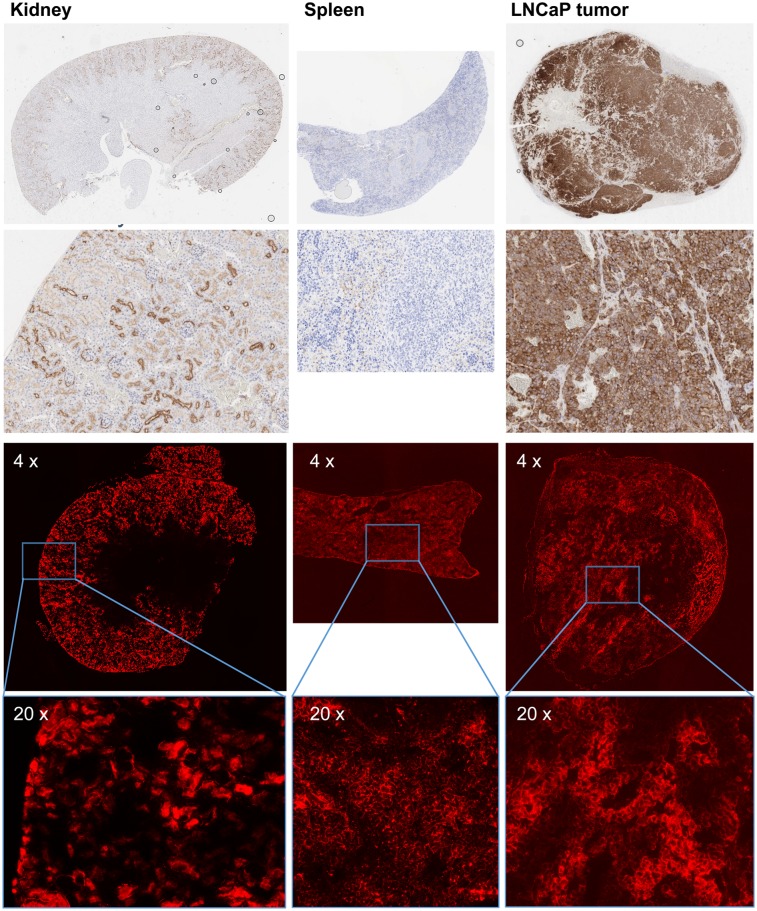
(Top 2 rows) PSMA immunohistochemistry of mouse tissue sections (paraffin embedded, 2 μm): first row, whole organ; second row, kidney and tumor 10× magnification and spleen 20× magnification. (Bottom 2 rows) Tissue distribution of PSMA-I&F (2 nmol, 1 h after injection) observed by Sulfo-Cy5 fluorescence microscopy of mouse tissue cryosections (10 μm): third row, 4× magnification; fourth row, 20× magnification. Please note that images have been scaled to comparable visual fluorescence intensity. Exposition times during fluorescence microscopy: 2 s for kidney, 12 s for spleen and tumor, respectively.

To investigate the dependence of PSMA-I&F uptake in different mouse organs (tumor, kidney, spleen) on in vivo PSMA expression, PSMA immunohistochemistry was performed on tissue samples of LNCaP xenograft–bearing CB17 SCID mice, and immunohistochemistry data were compared with the results obtained by fluorescence microscopy of tissue cryosections 1 h after injection of PSMA-I&F in the same tumor model (Fig. 5).

PSMA immunohistochemistry revealed intense and homogeneous membrane staining of all tumor cells in the LNCaP tumor xenograft, whereas spleen tissue was found to be essentially PSMA-negative. Only very weak membranous staining of single vascular endothelial cells as well as single lymphocytes within the red pulp of the spleen was observed. In the kidneys, intense PSMA staining was detected in parietal cells of the Bowman capsule and the proximal convoluted tubules (luminal membrane), alongside with gradually decreasing PSMA expression in the more distal parts of the renal tubules. The same distribution pattern with moderate to high locally restricted uptake of PSMA-I&F in the renal cortex was observed in fluorescence microscopy, indicating PSMA-specific accumulation in the tubules and glomeruli. However, in contradiction to the virtually nonexistent PSMA expression in the spleen, fluorescence microscopy revealed homogeneous membrane binding of PSMA-I&F to spleen tissue. Also in tumors, PSMA-I&F uptake did not consistently correlate with the immunohistochemistry data. Although PSMA expression was high throughout the entire tumor specimen, accumulation of PSMA-I&F was inhomogeneous. In contrast to the spleen, however, PSMA-I&F fluorescence was not only membrane-associated but also seemed to be internalized into tumor cells, as suggested by high cytosolic ligand uptake.

### Fluorescence-Guided Surgery

**FIGURE 6.**
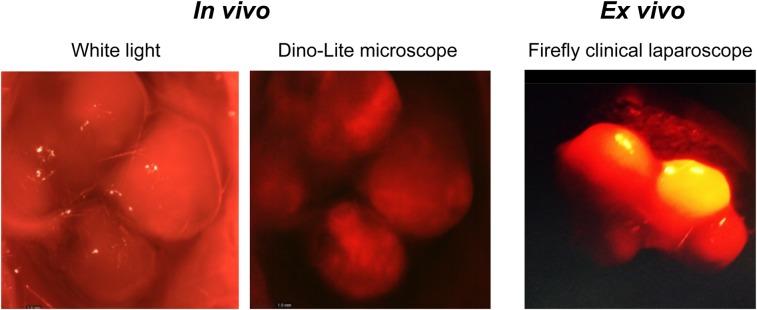
(Left) White light visualization and in vivo intraoperative fluorescence imaging of tumor in LNCaP xenograft–bearing CB17 SCID mouse 1 h after injection of 2 nmol PSMA-I&F. Sulfo-Cy5 fluorescence was detected using handheld Dino-Lite digital microscope. (Right) Ex vivo Sulfo-Cy5 fluorescence imaging of same (excised) LNCaP tumor using a clinical Firefly laparoscope.

To demonstrate the suitability of PSMA-I&F as a targeted probe for the intraoperative detection and fluorescence-guided resection of PSMA-expressing prostate carcinoma tissue, and to investigate the general compatibility of a Cy5-conjugated fluorescent probe with clinically used fluorescence cameras such as the Firefly laparoscope, proof-of-concept intraoperative and ex vivo fluorescence imaging using PSMA-I&F was performed in an LNCaP tumor–bearing mouse (Fig. 6). Both experimental settings allowed the sensitive in vivo detection of the PSMA-I&F–positive tumor xenograft with good contrast to the surrounding tissue.

## DISCUSSION

The results obtained in the preclinical evaluation of the novel PSMA-targeted hybrid compound ^68^Ga/^177^Lu-PSMA-I&F supply convincing evidence for the validity of the major working hypothesis, on which the design of this proof-of-concept compound was based, that is, that the PSMA-I&T scaffold is a versatile PSMA-targeted molecular platform for the generation of structurally diverse nuclear and hybrid probes.

This is primarily supported by 2 findings: the nearly identical PSMA-targeting efficiency observed for PSMA-I&F and its ^68^Ga- and ^177^Lu-labeled analogs compared with the respective PSMA-I&T reference compounds (Fig. 1; Table 1), both in vitro and in vivo, and the comparably small effect of linker extension and dye conjugation on the pharmacokinetics and in vivo performance of PSMA-I&F.

In contrast, for other structurally similar hybrid PSMA inhibitors such as ^111^In-DOTA-Lys(N_ε_-IRDye800CW)-Sub-KuE (31) or IRDye800CW-^68^Ga-HBED-CC-KuE (32), both dye conjugation and metal chelation were found to have considerable impact on PSMA affinity or internalization efficiency as well as in vivo biodistribution and clearance kinetics. Although for ^111^In-DOTA-Lys(N_ε_-IRDye800CW)-Sub-KuE, PSMA affinity was found to remain essentially unaffected by dye conjugation (10,31), its background clearance was substantially delayed compared with the parent compound ^68^Ga-DOTA-Lys-Sub-KuE (10), as indicated by a 5-fold increased blood activity concentration at 1 h after injection. Consequently, tracer uptake in the excretion organs and the tumor xenograft (PC-3 PIP) were also increased by a factor of 2–4.

In the case of the fluorescently labeled ^68^Ga-HBED-CC-KuE analogs (32), conjugation with near-infrared dyes led to a loss in PSMA affinity, but also an unexpected increase in internalization efficiency. Again, tracer clearance from the circulation and from the background was markedly delayed, but accumulation in the LNCaP tumors was also 3-fold increased.

These effects were not observed for ^68^Ga-PSMA-I&F. In our study, attachment of Sulfo-Cy5 conveyed a slight increase in lipophilicity and plasma protein binding (Table 1), leading to slightly delayed clearance kinetics of ^68^Ga-PSMA-I&F compared with ^68^Ga-PSMA-I&T. Only a negligible effect on tracer accumulation in the excretion organs was observed (Table 2), ultimately providing interchangeable PET imaging results using ^68^Ga-PSMA-I&F (Fig. 3) and ^68^Ga-PSMA-I&T (4).

Interestingly, although the observed effects of the respective fluorescent dye on the PSMA-targeting, clearance kinetics, and biodistribution pattern of ^111^In-DOTA-Lys(N_ε_-IRDye800CW)-Sub-KuE, IRDye800CW-^68^Ga-HBED-CC-KuE, and ^68^Ga-PSMA-I&F are fundamentally different, the tumor-to-background ratios for the 3 compounds were similar (tumor-to-blood, tumor-to-liver, tumor-to-intestines, and tumor-to-muscle: 1.4, 5.8, 7.7, and 11.6 [^111^In-DOTA-Lys(N_ε_-IRDye800CW)-Sub-KuE (31)]; 4.5, 4.9, 5.7, and 4.8 [IRDye800CW-^68^Ga-HBED-CC-KuE (32)]; and 2.1, 5.2, 9.6, and 9.6 [^68^Ga-PSMA-I&F], respectively).

Overall, however, using the PSMA-I&T scaffold in combination with the comparably small Sulfo-Cy5 dye had the distinct advantage of providing PSMA-I&F as a hybrid probe with reliable PSMA-targeting properties and a suitable biodistribution pattern. Additionally, the general utility of the far-red dye Sulfo-Cy5 for sensitive ex vivo and in vivo fluorescence imaging was supported by the imaging data obtained with PSMA-I&F (Figs. 4–6). Particularly noteworthy in this context is the compatibility of Sulfo-Cy5 not only with dedicated preclinical fluorescence cameras, but also with the clinical Firefly laparoscope (Fig. 6), which was able to detect the PSMA-I&F–accumulating LNCaP xenograft with high sensitivity. With more than 4,000 installs of surgical robots equipped with this camera (Intuitive Surgical Inc.; https://www.intuitive.com/) worldwide, the general translational potential of Sulfo-Cy5–conjugated targeted hybrid probes for intraoperative guidance is thus greatly enhanced.

A relatively surprising result from our fluorescence microscopy studies (Fig. 5) was the limited accordance of PSMA-I&F accumulation in the tumor, kidneys, and spleen with the corresponding PSMA expression level. Excellent colocalization of focal tubular PSMA expression and PSMA-I&F uptake was detected only in the kidneys. These data accurately reflect the human situation, where endogenous tubular PSMA expression (47) is primarily held responsible for the generally high renal uptake of PSMA-targeted radiotracers. However, given the comparably low overall renal PSMA expression compared with tumor (Fig. 5), the observed greater than 20-fold-higher (and only partly blockable [Fig. 3]) uptake of ^68^Ga-PSMA-I&F in the kidney than in the LNCaP xenograft is strongly indicative of alternative, non–PSMA-mediated uptake mechanisms, such as megalin/cubilin-mediated tubular reabsorption (48), being involved in the renal handling of PSMA-targeted tracers.

In the tumor, tracer uptake was substantially lower and less homogeneous than what would have been expected from the consistently high PSMA expression throughout the entire specimen. Here, limited perfusion of the fast-growing xenograft seems the most probable reason for the observed inhomogeneous PSMA-I&F uptake.

For the spleen, the most contradictory results were obtained. The observed absence of PSMA expression in mouse spleen is in agreement with data from the literature, reporting PSMA expression in mouse spleen on the messenger RNA level (49) but not on the protein level (50). However, homogeneous membrane binding of PSMA-I&F was observed, resulting in the observed high uptake of ^68^Ga-PSMA-I&F in the spleen (Table 2). Thus, although being efficiently blockable by an excess of 2-PMPA (20), the splenic uptake of PSMA-I&T–based ligands in mice seems to be mediated by a PSMA-independent process. Further studies, also with respect to the significance of these observations for the human situation, are required.

## CONCLUSION

The preclinical evaluation of PSMA-I&F and its ^68^Ga- and ^177^Lu-labeled analogs has conclusively demonstrated the general feasibility of a PSMA-I&T–based hybrid tracer concept using the far-red fluorescent dye Sulfo-Cy5. Despite substantial structural changes compared with the parent compound ^68^Ga/^177^Lu-PSMA-I&T, ^68^Ga/^177^Lu-PSMA-I&F maintains an unchanged, high PSMA-targeting efficiency and a favorable pharmacokinetic profile, allowing for sensitive and high-contrast detection of PSMA expression in vivo via nuclear (PET/SPECT) and optical imaging methods (intraoperative imaging/in vivo and ex vivo fluorescence microscopy). Thus, the PSMA-I&T scaffold has once more been shown to represent a versatile PSMA-targeted molecular platform, which allows relatively straightforward adaptation to the different structural requirements of dedicated nuclear or hybrid imaging agents.

## DISCLOSURE

This study was financially supported by the Deutsche Forschungsgemeinschaft (SFB824; subprojects Z1 and Z3). Hans-Jürgen Wester is founder and shareholder of Scintomics GmbH. Vasilis Ntziachristos serves as consultant for SurgVision BV, and Maximilian Koch is an employee of Bracco Imaging Germany GmbH. No other potential conflict of interest relevant to this article was reported.
