# Supplementary material for: Synthesis and Preclinical Characterization of the PSMA-Targeted Hybrid Tracer PSMA-I&F for Nuclear and Fluorescence Imaging of Prostate Cancer
Source: J Nucl Med. 2019 Jan;60(1):71–8. doi: 10.2967/jnumed.118.212720 (PMC6354225; doi:10.2967/jnumed.118.212720)
Supplement: Supplementary file 1 [file jnm212720SupplementalData.pdf]

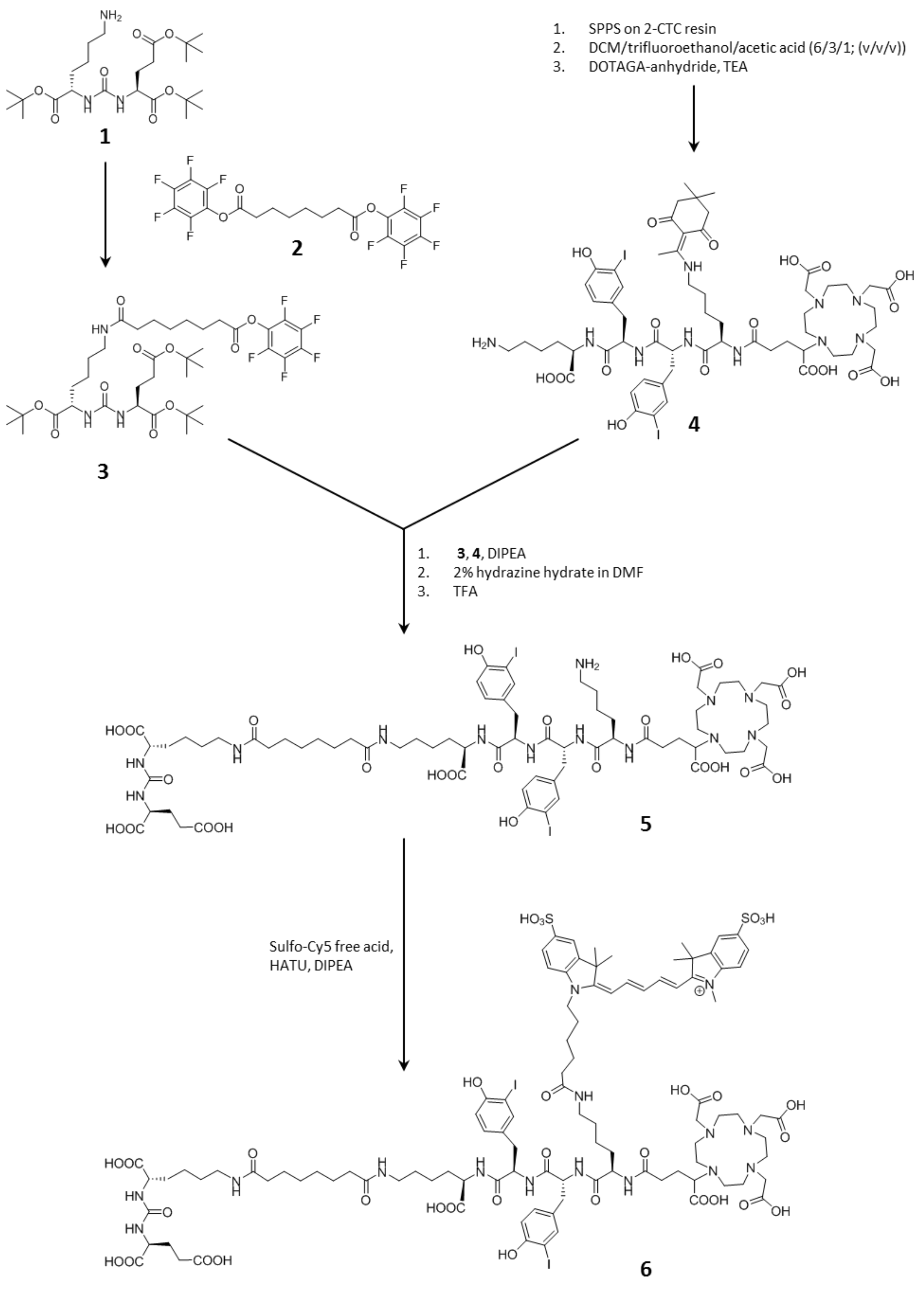

Supplemental Fig. 1 *Synthesis Scheme for PSMA-I&F (**6**)*

Ex vivo Evaluation

Whole-body cryosectioning and fluorescence imaging

A LNCaP xenograft bearing SHO mouse (the same animal that had been used for the static PET scan two days previously) was injected with 2 nmol of ^{nat}Ga-PSMA-I&F, sacrificed at 1h p.i., embedded in a mixture of Tissue-Tek and black ink (7.41% v/v) inside a cylinder of 3.5 cm diameter, and placed into the -80°C freezer overnight. Before sectioning the specimen was placed into the cryostat at -17°C for two hours.

Cryosections were prepared using a Leica CM 3500 cryostat (Leica, Wetzlar, Germany) at a slice thickness of 20 µm. The temperature during slicing was adjusted to -17°C. A highly sensitive fluorescence imaging system was mounted onto the cryostat and imaged the specimen after every 4 slices. The imaging protocol consisted of the acquisition of three reflectance images at 630/60 nm (red channel), 535/38 nm (green channel), and 460/50 nm (blue channel) for color imaging and one fluorescence image at 670/10 nm, which is close to the maximum emission of the sulfo-Cy5 (i.e. 662 nm). We employed a halogen light source (KL2500, Schott, New York, USA) coupled to fiber bundles and a motorized filter-wheel (FW102C, Thorlabs, New Jersey, USA) to illuminate the specimen. Reflectance images were enabled by white-light illumination (i.e. no light source filtering), whereas bandpass filter (ET620/60X, Chroma Technology, Rockingham, Vermont, United States) was placed in the filter-wheel to enable sulfo-Cy5 excitation.

Images were recorded by an iXon electron multiplying charge-coupled device (EMCCD, DV8201-BV, Andor Technology, Belfast, Northern Ireland). A Leica Z16 Apo Macroscope was coupled to the camera, allowing manual adjustments of zoom, focus, and fields of view (FOV). In the lens-camera path a 25-positions filter-wheel was placed with mounted three bandpass filters (D630/60M, D535/38M, and D460/50X, Chroma Technology) for reflectance acquisition and one bandpass filter (D670/10X, Chroma Technology) for fluorescence acquisition. Supp. Fig. 1 depicts a schematic of the system.

The serial sectioning and imaging system was fully automated using custom software implemented in LabView (National Instruments, Austin, USA) to control sectioning and trigger image acquisition (4). Gain of the EMCCD was maintained constant during sectioning at 2, 10 and 20 for the red, green and blue

channels, and at 300 for fluorescence imaging, while exposure time was automatically adjusted per imaged slice and ranged from 0.001 s to 20 s depending on the per slice amount of the reflected or emitted light. Post-processing involved normalization of the acquired data to their corresponding exposure time, reconstruction of the color images from the red, green, and blue channels, and longitudinal alignment of the three-dimensional stack of imaged slices for optimal resolution. These processes were implemented in MATLAB (Mathworks, Natick, USA), while Amira (FEI Visualization Sciences Group, Burlington, USA) was used for cross-sectional and longitudinal visualization of the acquired data, and three-dimensional representation of the fluorescence data.

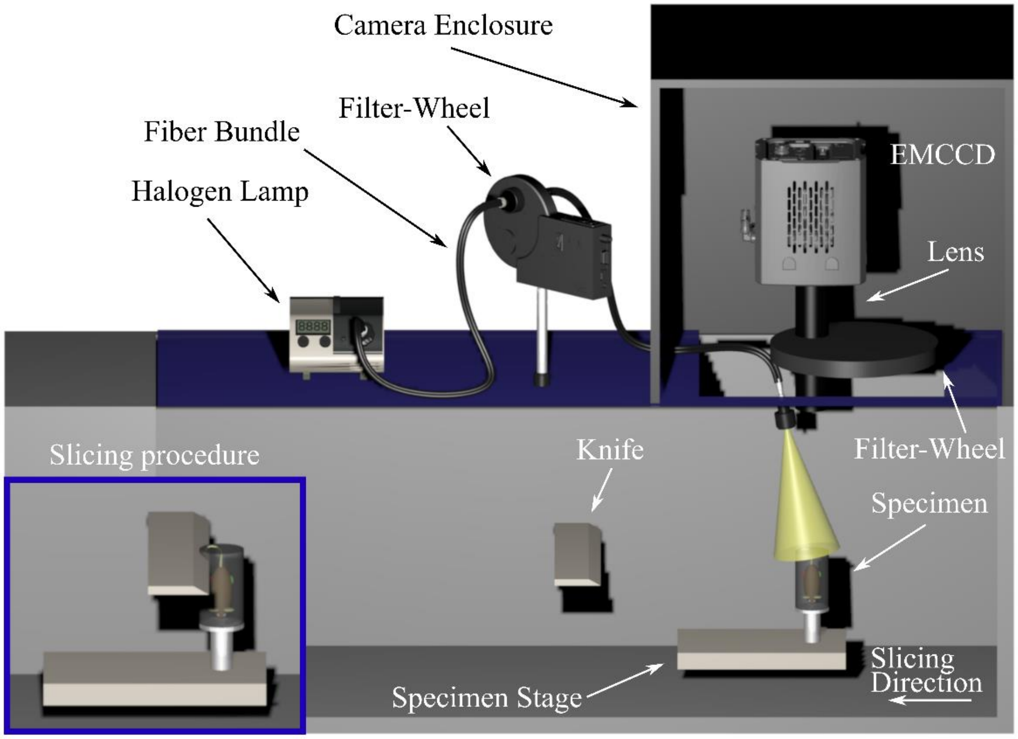

Supplemental Fig. 2. *The serial sectioning and imaging system. A halogen lamp is employed to deliver illumination onto the imaged specimen. Reflectance and fluorescence images are acquired by a highly sensitive electron multiplying charge-coupled device (EMCCD) at specific spectral bands defined by filter-wheels at both the excitation and detection illumination paths. Left inset depicts the slicing procedure, which is repeated 4 times with a step of 20 μm before imaging the sample (resulting to 80 μm imaging step). All CAD optomechanical components are courtesy of Thorlabs (www.thorlabs.com).*

RESULTS

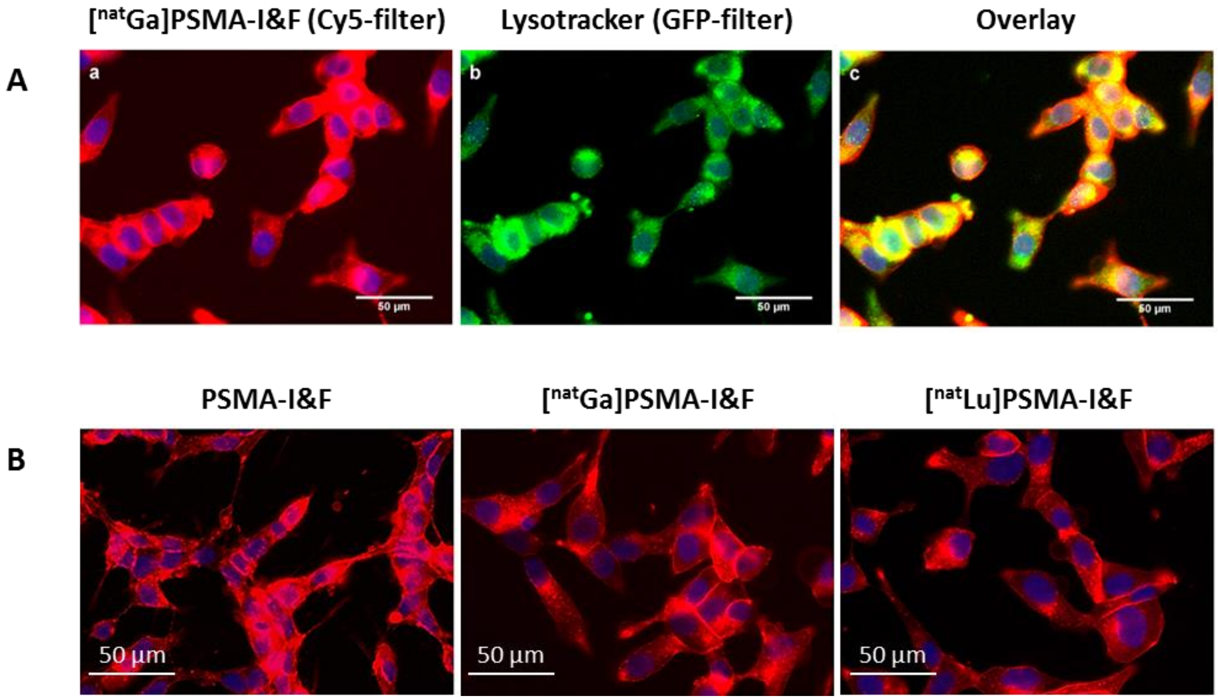

Supplemental Fig. 3 ***A)*** *Fluorescence microscopy of the internalization of [natGa]PSMA-I&F (100 nM) into LNCaP prostate carcinoma cells after 60 min at 37°C (a) (Cy5-filter; overlay with nuclear blue fluorescence acquired using the DAPI filter (Hoechst 33342)). Images acquired using the GFP filter show uptake of the lysosomal dye LysoTracker (b). An overlay of all three channels is shown on the right (c).*
B) *Fluorescence microscopy of the internalization of PSMA-I&F (free DOTAGA), [natGa]PSMA-I&F and [natLu]PSMA-I&F (25 nM) into LNCaP prostate carcinoma cells after 60 min at 37°C.*

1. Weineisen M, Simecek J, Schottelius M, Schwaiger M,Wester HJ. Synthesis and preclinical evaluation of DOTAGA-conjugated PSMA ligands for functional imaging and endoradiotherapy of prostate cancer. *EJNMMI research.* 2014;4:63.
2. Robu S, Schottelius M, Eiber M, Maurer T, Gschwend J, Schwaiger M, et al. Preclinical Evaluation and First Patient Application of 99mTc-PSMA-I&S for SPECT Imaging and Radioguided Surgery in Prostate Cancer. *J Nucl Med.* 2017;58:235-242.
3. Breeman WA, De Jong M, Visser TJ, Erion JL,Krenning EP. Optimising conditions for radiolabelling of DOTA-peptides with 90Y, 111In and 177Lu at high specific activities. *Eur J Nucl Med Mol Imaging.* 2003;30:917-920.
4. Symvoulidis P, C. C. Perez, Schwaiger M, Ntziachristos V,Westmeyer GG, Serial sectioning and multispectral imaging system for versatile biomedical applications. In *2014 IEEE 11th International Symposium on Biomedical Imaging (ISBI)*, 2014; pp 890-893.
